# Processing Technology Selection for Municipal Sewage Treatment Based on a Multi-Objective Decision Model under Uncertainty

**DOI:** 10.3390/ijerph15030448

**Published:** 2018-03-05

**Authors:** Xudong Chen, Zhongwen Xu, Liming Yao, Ning Ma

**Affiliations:** 1 College of Management Science, Chengdu University of Technology, Chengdu 610059, China; chenxudong198401@163.com; 2 Business School, Sichuan University, Chengdu 610065, China; xuzhongwen1996@163.com (Z.X.); ningma_610@163.com (N.M.)

**Keywords:** processing technology selection, municipal sewage treatment, multi-objective decision

## Abstract

This study considers the two factors of environmental protection and economic benefits to address municipal sewage treatment. Based on considerations regarding the sewage treatment plant construction site, processing technology, capital investment, operation costs, water pollutant emissions, water quality and other indicators, we establish a general multi-objective decision model for optimizing municipal sewage treatment plant construction. Using the construction of a sewage treatment plant in a suburb of Chengdu as an example, this paper tests the general model of multi-objective decision-making for the sewage treatment plant construction by implementing a genetic algorithm. The results show the applicability and effectiveness of the multi-objective decision model for the sewage treatment plant. This paper provides decision and technical support for the optimization of municipal sewage treatment.

## 1. Introduction

With the continuous advancement of China’s economic construction and the enhancement of an overall social consciousness regarding environmental protection, municipal sewage treatment, known as an important measure to protect water resources and control water pollution, has received increasing attention. Many studies have shown that municipal sewage contains large amounts of contaminants, such as nitrogen and phosphorus [1], resistance genes and hormones [2,3] and trace contaminants [4,5]. Several countries in Europe and the United States have constructed many municipal sewage treatment plants, which have effectively controlled environmental water pollution [6,7]. However, in some of the sewage treatment plants, especially the county-level sewage treatment plants, there is a lack of funds for their operation [8]. In addition, with the annual increase in municipal sewage treatment capacity, a dramatic increase in municipal sewage sludge production has occurred, with concomitant concerns regarding improper sludge disposal and retention of organic contaminants [9]; therefore, secondary pollution to the water and the atmosphere will occur if the sludge enters the environment without the appropriate disposal measures [10]. Hence, sludge should be considered from a comprehensive perspective. In China, water resources per capita are scarce, especially in the densely populated cities, and water pollution is more serious [11]. Unfortunately, there are also some existing backwards for municipal sewage treatment, such as high operation costs and low removal efficiency [12]. Therefore, in order to align with the sustainable development of society, it is necessary to analyze the overarching factors, such as technology, economy, environment and management in relation to sewage treatment and put forward scientific suggestions for the construction of sewage treatment plants.

Processing technology is the most important factor affecting sewage treatment, and mainstream sewage treatment processes rely on biological treatment methods because of technical and cost efficiencies [13,14], which mainly include the activated sludge method and the biofilm method. In addition, the activated sludge method is more widely used because of its sewage removal capacity [15], including Intermittent Cycle Extended Aeration System (ICEAS) process, Anaerobic Anoxic Oxic (AAO) method, and oxidation ditch. Bhatnagar and Sillanpää, [16] analyzed the application of adsorbent in sewage treatment. Kartal et al. [17] claimed that the use of anoxic ammonium-oxidizing (anammox) bacteria can facilitate effective removal of ammonia and nitrite. Plakas and Karabelas [18] argued that nanofiltration (NF) and low-pressure reverse osmosis (LPRO) can be used to treat pesticides in sewage. Xu et al. [19] and Fu et al. [20] summarized the application of zero-valent iron (ZVI), including nanoscale zero-valent iron (nZVI)), in wastewater treatment.

In addition to the processing technology, there are many factors that will affect the sewage treatment plant construction program design, such as construction scale and site selection of the sewage treatment plant [21,22]. Hernandez-Sancho et al. [21] considered the impact of environmental and economic benefits on the design of wastewater treatment plants. Cornel and Schaum [23], Bresler [24] and Dodane [25] focused on the impact of costs in the construction of wastewater treatment plants, which was related to a number of factors [26]. Molinos-Senante et al. [27] considered a cost-benefit analysis of wastewater treatment plants from an economical perspective. In addition, to consider the impact of the size of the sewage treatment plant on the cost, Hernandez-Sancho et al. [28] also considered the impact of the pollutant removal rate and the equipment age. Mojahed et al. [29] analyzed the planning of wastewater treatment plants under environmental control. There are also some studies that analyzed the construction of sewage treatment plants from the life cycle assessment (LCA) perspective [30,31,32]. In all, there are many factors that affect the design of municipal sewage treatment, such as processing technology, site location, environmental protection, investment costs and operational efficiency. Through a review of the above literature, it can be deduced that existing municipal sewage treatment facilities lack an important single factor, namely, a systematic theoretical research approach; moreover, the research conclusion is often not very practical. Hence, faced with these five indicators in terms of environmental protection and economic benefit, a multi-objective programming approach is more suitable [33].

This paper aims to put forward some scientific insights regarding the construction of sewage treatment plants by developing a multi-objective decision model that is subject to some relevant constraints, such as pollutant control capacity, etc. In addition, the contributions of this paper are summarized as follows:

1. By combining multiple factors influencing the construction of sewage treatment plants, this study considers the trade-offs between environmental protection and economic benefit.
2. A general model of multi-objective decision-making under uncertainty conditions is proposed for optimizing the problem of municipal sewage treatment plant construction.

The structure of this paper is as follows: The introduction provides reviews of previous academic research in the area of urban sewage treatment planning. A multi-objective decision model to solve the urban sewage treatment plan problem is established in Section 2. Section 3 is dedicated to application research. Section 4 discusses conclusions drawn from the research.

## 2. Description of Multi-Objective Decision Model Building

### 2.1. Key Problem Statement

The treatment of municipal sewage has become one of the essential measures to protect water resources and to control water pollution. Nevertheless, the operation of current municipal sewage treatment plants is not promising. A large number of the sewage treatment plants are not open throughout the entire year because of high operation costs, weak technical strength, inadequate staff ability and so on. Therefore, some plants fail to operate at full capacity; moreover, China’s urban sewage treatment facilities seriously lag more robust implementations and are inadequate [34], leading to a poor return on this portion of the national investment. Hence, a comprehensive analysis of the factors impacting the municipal sewage treatment plants from the perspective of systems engineering is needed, which can deliver more acceptable solutions that address the perspectives of the stakeholders [35]. 

According to the reports of relevant departments, there are multiple reasons for these phenomena, for instance, unclear operational costs, weak technical strengths, inadequately trained staff, poor operations management and design problems.

#### 2.1.1. Principal Factors

Sewage treatment technology is the most important factor affecting the results of the sewage treatment. A suitable processing technology is important and should be chosen according to the actual local water requirements. Given the different qualities of municipal sewage and domestic sewage, different processing technologies are required. The primary technologies used for municipal sewage treatment in China are the Sequencing Batch Reactor (SBR) process, the oxidation ditch process and the traditional activated sludge process. Each technology for sewage treatment has its advantages, characteristics, applicable conditions, and deficiencies.

Additionally, the site selection has influences on the sewage treatment plants. The optimal site of a sewage treatment plant is critical because an optimally established wastewater treatment plant can efficiently protect the environment and enable the sustainability of economic and urban development. Moreover, the control target of pollutant discharge, the urban geographic and geological environment, the function and flow volume of the receiving water and land type and quality are factors that require consideration.

#### 2.1.2. Environmental Impacts and Economic Benefits

Environmental impacts and economic benefits are of equal importance. From the perspective of environmental protection, the construction of the sewage treatment plants generally should not cause irreparable damage to the surrounding environment. Therefore, unsuitable locations include upwind of a city, upstream of an urban water source or in close proximity to residential areas. After the sewage treatment plant is put into operation, its effects should be minimized on the environmentally sensitive areas, the downstream areas for water conservation and, in particular, the aquaculture at the towns. Moreover, the impact should not exceed the local environmental capacity.

From the perspective of economic benefits, the reuse of wastewater is becoming an essential approach to addressing water shortages. After the treated sewage reaches the prescribed standard, it can be used as irrigation water, industrial water, and domestic water for toilet flushing, garden watering and road cleaning. Therefore, the benefits of return water utilization can be considered when optimizing the construction scheme.

#### 2.1.3. Uncertainty Problem

**Figure 1:**
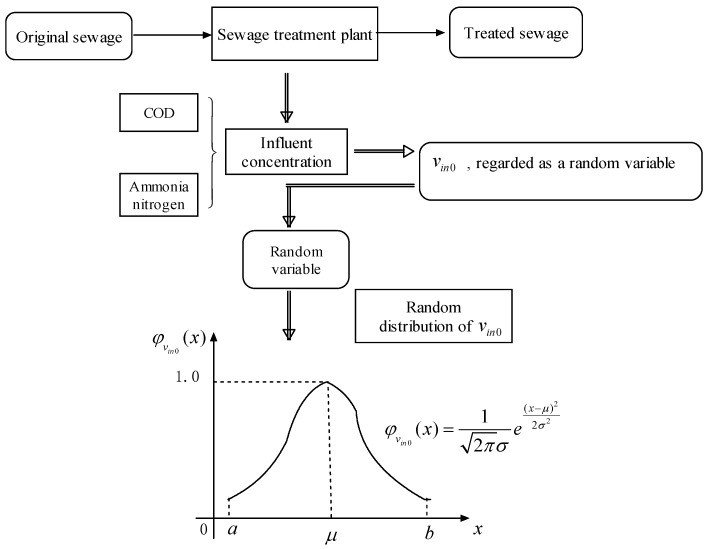
Uncertainty of urban sewage treatment problem.

Uncertainty widely exists in various social phenomena, natural phenomena and engineering practice. As a complex system, the municipal sewage treatment system includes three kinds of elements: human, matter and environment, and has three key processes: input, inner system and output, so it includes various kinds of uncertainty (see Figure 1). 

First, inherent uncertainties in nature include hydrology, geography, temperature, precipitation, and amount of solar radiation, and the environmental parameters can constantly change with the changes in the weather and other conditions. In a sewage treatment system, the condition of the original sewage input, the treatment effectiveness and the environmental influence of the treated sewage output are subject to uncertainty due to changes in such parameters as temperature, humidity, and radiation exposure. For example, the input volume is highly related to the temperature and rainfall; the treatment effectiveness is related to the temperature, light and humidity. Second, the uncertainties caused by human activity in a sewage treatment system are mainly reflected in the input and treatment processes. For, example, the releasing of water saving policies could decrease the original sewage volume; domestic sewage is greater on non-working days and industrial sewage is greater on working days. Meanwhile, possible sudden emergencies such as a pipeline leakage may increase the pollutant level in the sewage. Additionally, the serious working attitude of the staff in the treatment plant has a positive influence on the effectiveness of sewage treatment. Third, the uncertainties caused by an engineering system can be reflected in the input and treatment processes. In the input process, the pollutant level of the original sewage may decrease with technological improvements adopted by the factories. In the treatment process, operational mistakes in the sewage treatment or monitoring deviations in sewage detection can negatively impact sewage treatment. Therefore, using a systems perspective, the sewage treatment problem has uncertainty in many aspects of its subsystems, and these characteristics facilitate using an uncertainty framework to solve this problem within the context of its environment. 

The sewage concentration is the most important element that is related to the decision of each process step and the final goals. The sewage influent concentration may influence the site and technology selections of a treatment plant and further influence the economic and ecological goals. The influent concentration involves the rate of the pollutants quantity and sewage volume, which is affected by various uncertain factors. Therefore, the influent concentration is also uncertain and difficult to define with crisp values. In this paper, the influent concentration is valued by two indices: Chemical Oxygen Demand (COD) $\nu_{in0}^{\alpha }$ and ammonia nitrogen $\nu_{in0}^{\beta }$ [12,36]. The corresponding managers can only predict the most possible value of influent concentration $\nu_{in0}$ which approximately follows a normal distribution, i.e., $\nu_{in0}\sim N\left(\mu ,\delta^{2}\right)$. Therefore, it is appropriate to consider a sewage treatment system as a random environment.

**Figure 2:**
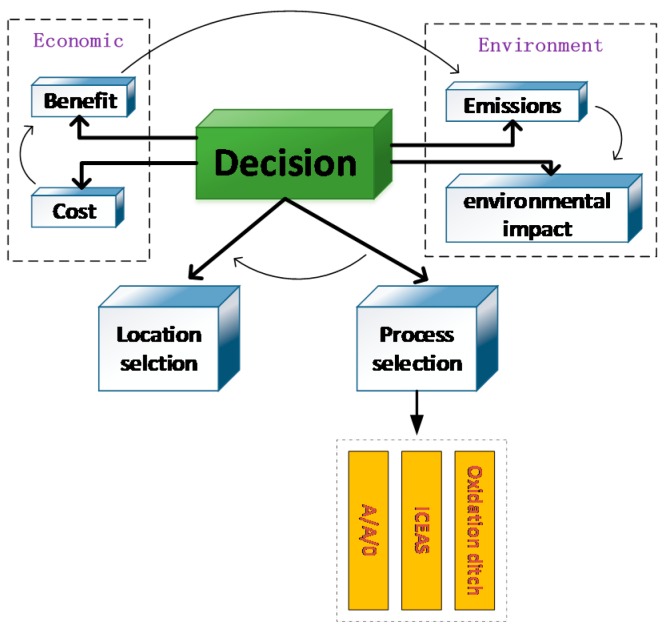
Economic-environment trade-off problem.

Using the background of uncertainty, factors such as the site selection, selection of wastewater treatment technology, environmental impacts and economic benefits allow for the development of a scientific construction plan for the sewage treatment plant. The uncertainty approach ensures the quality of sewage treatment, guarantees the stable operation of the plant and meets the treatment standards. Hence, the construction scheme is the focus of this paper (see Figure 2).

### 2.2. Question Assumption

1. All construction schemes are aligned with the overall land utilization planning, urban master planning, special planning of a sewage treatment, as well as such regulations as relevant laws, specifications, and procedures.
2. Due to the constraints of factors such as the floor space, the paper assumes that in terms of decision-making, the number of sewage treatment plants to be built is less than *N*.
3. It is assumed that the distributions of the main sewage source at the construction site of a treatment plant is determined and assumed that the influent concentrations are the same.
4. Apart from the infrastructure investments affected by the design treatment capacity and efficiency, the construction and investment costs of a sewage treatment plant also include the purchase and installation costs of different pieces of treatment equipment required by different processes.
5. The management and operation costs mainly consist of, for instance, pipeline maintenance, energy consumption, and equipment repair, which is different from sewage treatment engineering.

In terms of the impact of a sewage treatment plant on the environment, the impact of the noise and odor of that plant on the surrounding residential land and sensitive spots are principally considered. Taking all of the environmental impact factors into account was accomplished by utilizing the fuzzy multi-attribute method; the larger the value, the greater the impacts of the site on the environment. 

### 2.3. Modeling

Based on the general multi-objective, decision-making model of a sewage treatment plant and considering the specific conditions of this case, this section establishes the objective functions and constraint conditions from the perspective of a cost model, eventually forming the multi-objective programming model which is suitable for this case.

#### 2.3.1. Objective Function

There are multiple factors affecting the construction of a sewage treatment plant, for instance, the overall consideration of the economic, social and environmental benefits, etc. This paper sets four objectives, namely, minimum cost, minimum environmental impact, maximum benefit of recycled water and maximum sewage treatment efficiency.

*x_i_* represents whether the optional site A*_i_* can be selected to build a sewage treatment plant: if *x_i_* = 1, then, site A*_i_* can be chosen to build a treatment plant. If *x_i_* = 0, then, site *A_i_* is not suitable to build such a plant. *y_ij_* denotes whether the *j* technology *T_j_* can be used in treatment plant *i*: if *y_ij_* = 1, then, *T_j_* can be used in the treatment plant *A_i_*. If *y_ij_* = 0, then, *T_j_* is not suitable for plant *A_i_*. The treatment capacity of technology *j* is denoted *t_j_*.

According to Assumption 4, the initial capital cost comprises construction cost of a treatment plant, cost of off-site sewage interception pipelines, land expropriation cost and household demolition compensation cost. Hence, the initial capital cost is:

(1) $$\sum_{i=1}^{n}C_{i}(Q_{i},y_{ij})x_{i}+\sum_{i=1}^{n}L_{i}(l_{i})x_{i}+\sum_{i=1}^{n}x_{i}D_{i}(s_{i})$$

According to Assumption 5, the management operation cost is:

(2) $$\sum_{i=1}^{n}x_{i}F_{i}(y_{ij})$$

Then, the objective function I pertains to the minimum investment cost, which can be written as:

(3) $$\min f_{1}(x_{i},y_{ij})=\sum_{j=1}^{m}\{\sum_{i=1}^{n}C_{i}(Q_{i},y_{ij})x_{i}+\sum_{i=1}^{n}L_{i}(l_{i})x_{i}+\sum_{i=1}^{n}x_{i}D_{i}(s_{i})+\sum_{i=1}^{n}x_{i}F_{i}(y_{ij})\}$$

The objective function II pertains to the minimum environmental impact, which can be written as:

(4) $$\min f_{2}\left(x_{i}\right)=\sum_{i=1}^{n}\delta_{i}x_{i}$$

Accordingly, the reclaimed sewage benefit of treatment plant *i* is the recycled water income $B_{i}\lambda_{i}Q_{i}$; therefore, the objective function III pertains to the maximum benefit, which can be written as:

(5) $$\max f_{3}(x_{i})=\sum_{i=1}^{n}B_{i}\lambda_{i}Q_{i}x_{i}$$

Upon completion of a treatment plant, the sewage treatment efficiency is expected to be maximized; the objective functions IV and V, therefore, are the minimum discharge of COD and ammonia nitrogen, respectively:

(6) $$\min f_{4}(x_{i},y_{ij})=E\left[\sum_{i=1}^{n}Q_{i}x_{i}(1-\lambda_{i}){\tilde{v}}_{outi}^{\alpha }\right]$$

(7) $$\min f_{5}(x_{i},y_{ij})=E\left[\sum_{i=1}^{n}Q_{i}x_{i}(1-\lambda_{i}){\tilde{v}}_{\mathrm{out}i}^{\beta }\right]$$

where $v_{outi}^{\alpha }=v_{in0}^{\alpha }-t_{j}y_{ij};v_{outi}^{\beta }=v_{in0}^{\beta }-t_{j}y_{ij}$.

#### 2.3.2. Constraints

1. Pollutant processing capacity. The pollutant processing capacity of a sewage treatment plant should not be less than the sewage quantity, and the actual processing capacity should be more than 60% of the design capacity. In other words:
  (8) $$0.6\sum_{i=1}^{n}Q_{i}x_{i}\leq \sum_{i=1}^{m}q_{i}\leq \sum_{i=1}^{n}Q_{i}x_{i}$$
2. Construction investment limitation. Before constructing a sewage treatment plant, the planning department sets a certain total investment; in other words, the construction investment funds should not exceed the upper limit of the total investment. It can be written as:
  (9) $$\sum_{i=1}^{n}C_{i}(Q_{i},y_{ij})x_{i}+\sum_{i=1}^{n}L_{i}(l_{i})x_{i}+\sum_{i=1}^{n}x_{i}D_{i}(s_{i})\leq M$$
  where *M* is the prescribed investment limit.
3. Emissions standards. According to the total pollutant control target, after completion of a sewage treatment plant, effluent must meet certain standards; in other words, the main pollutant content should be less than the standard content. It can be written as:
  (10) $$\mathrm{COD}:\;E\left[\sum_{i=1}^{n}Q_{i}x_{i}(1-\lambda_{i})({\tilde{v}}_{in0}^{\alpha }-t_{j}y_{ij})\right]<W_{\mathrm{COD}},$$
  (11) $${\mathrm{NH}}_{3}-NE\left[\sum_{i=1}^{n}Q_{i}x_{i}(1-\lambda_{i})({\tilde{v}}_{in0}^{\beta }-t_{j}y_{ij})\right]<W_{{\mathrm{NH}}_{3}-N},$$
  where *W*_COD_ and $W_{{\mathrm{NH}}_{3}-N}$ represent COD and ammonia nitrogen emissions control standards of that region, respectively.
4. Effluent concentration limitation. To meet discharge standards, effluent concentration should be less than the upper limit of the stated standard. That is:
  (12) $$E\left[v_{in0}^{\alpha }-t_{j}y_{ij}\right]<\eta_{0}^{\alpha },i=1,2,\ldots ,n$$
  (13) $$E\left[v_{in0}^{\beta }-t_{j}y_{ij}\right]<\eta_{0}^{\beta },i=1,2,\ldots ,n$$
  where $\eta_{0}^{\alpha }$ and $\eta_{0}^{\beta }$ denote the stated upper limits of wastewater COD and ammonia nitrogen concentration, respectively. Pollutant discharge standards are generally divided into three types: Primary standard A (COD, 50 mg/L; NH_3_-N, 8 mg/L), Primary standard B (COD, 60 mg/L, NH_3_-N, 15 mg/L) and secondary standard (COD, 100 mg/L; NH_3_-N, 25 mg/L).
5. Pollutant treatment rate limitation. The pollutant treatment rate should be more than the lowest limit:
  (14) $$E\left[\left(v_{in0}^{\alpha }-t_{j}y_{ij}\right)/v_{in0}^{\alpha }\right]>\theta_{0}^{\alpha },i=1,2,\ldots ,n$$
  (15) $$E\left[\left(v_{in0}^{\beta }-t_{j}y_{ij}\right)/v_{in0}^{\beta }\right]>\theta_{0}^{\beta },i=1,2,\ldots ,n$$
  where $\theta_{0}$ is the stated lower limit of the pollutant treatment rate.
6. Plant quantity limitation. The number of sewage treatment plants to be constructed should not exceed a certain limit:
  (16) $$\sum_{i=1}^{n}x_{i}\leq N$$
7. Processing technology selection limitation. Each wastewater treatment plant can only choose to use one kind of disposal process:
  (17) $$\sum_{j=1}^{m}y_{ij}=1,\;i=1,2,\ldots ,n$$

Based on the analysis above, the paper establishes the following multi-objective programming model subject to constraints (8)–(17):

(18) $$\begin{array}{l} \min f_{1}(x_{i},y_{ij})=\sum_{i=1}^{n}C_{i}(Q_{i},y_{ij})x_{i}+\sum_{i=1}^{n}L_{i}(l_{i})x_{i}+\sum_{i=1}^{n}x_{i}D_{i}(s_{i})+\sum_{i=1}^{n}x_{i}F_{i}(y_{ij})\\ \min f_{2}(x_{i})=\sum_{i=1}^{n}\delta_{i}x_{i}\\ \max f_{3}(x_{i},)=\sum_{i=1}^{n}B_{i}\lambda_{i}Q_{i}x_{i}\\ \min f_{4}(x_{i},y_{ij})=E\left[\sum_{i=1}^{n}Q_{i}x_{i}(1-\lambda_{i})({\tilde{v}}_{in0}^{\alpha }-t_{j}y_{ij})\right]\\ \min f_{5}(x_{i},y_{ij})=E\left[\sum_{i=1}^{n}Q_{i}x_{i}(1-\lambda_{i})({\tilde{v}}_{in0}^{\beta }-t_{j}y_{ij})\right]\\ s.t.\;\left\{ \begin{array}{l} 0.6\sum_{i=1}^{n}Q_{i}x_{i}\leq \sum_{i=1}^{m}q_{i}\leq \sum_{i=1}^{n}Q_{i}x_{i}\\ \sum_{i=1}^{n}C_{i}(Q_{i},y_{ij})x_{i}+\sum_{i=1}^{n}L_{i}(l_{i})x_{i}+\sum_{i=1}^{n}x_{i}D_{i}(s_{i})\leq M\\ E\left[\sum_{i=1}^{n}Q_{i}x_{i}(1-\lambda_{i})({\tilde{v}}_{in0}^{\alpha }-t_{j}y_{ij})\right]<W_{\mathrm{COD}}\\ E\left[\sum_{i=1}^{n}Q_{i}x_{i}(1-\lambda_{i})({\tilde{v}}_{in0}^{\beta }-t_{j}y_{ij})\right]<W_{{\mathrm{NH}}_{3}-N}\\ E\left[{\tilde{v}}_{in0}^{\alpha }-t_{j}y_{ij}\right]<\eta_{0}^{\alpha },\;i=1,2,\ldots ,n\\ E\left[{\tilde{v}}_{in0}^{\beta }-t_{j}y_{ij}\right]<\eta_{0}^{\beta },\;i=1,2,\ldots ,n\\ E\left[({\tilde{v}}_{in0}^{\alpha }-v_{\mathrm{out}i}^{\alpha })/{\tilde{v}}_{in0}^{\alpha }\right]>\theta_{0}^{\alpha },\;i=1,2,\ldots ,n\\ E\left[({\tilde{v}}_{in0}^{\beta }-v_{\mathrm{out}i}^{\beta })/{\tilde{v}}_{in0}^{\beta }\right]>\theta_{0}^{\beta },\;i=1,2,\ldots ,n\\ \sum_{i=1}^{n}x_{i}\leq N\\ \sum_{j=1}^{m}y_{ij}=1,\;i=1,2,\ldots ,n\\ x_{i},\;y_{ij}=0\;\mathrm{or}\;1 \end{array}\right. \end{array}$$

## 3. Application Research

The resident population of the urban area of *X* county, a Chengdu suburb in Sichuan Province, is 112,000. According to the county development plan, it will reach 150,000 by 2015. By reference to per capita household water consumption in Sichuan Province, which is 128.5 L/day, the total domestic water consumption of that area is approximately 1.9 × 10^4^ m^3^/day. By calculating on the basis of 90% of the predicted total domestic water consumption, household sewage discharge will be 1.7 × 10^4^ m^3^/day. In addition to sanitary sewage, due to a large number of textile mills and garment factories in the county, the industrial water consumption is 1.5 × 10^4^ m^3^/day. By calculating on the basis of 0.7 of the design coefficient, the predicted industrial wastewater discharge is approximately 1.1 × 10^4^ m^3^/day. Therefore, the recent overall sewage discharge of that county will be 2.8 × 10^4^ m^3^/day, 60.7% of which is sanitary sewage.

Because of rapid industrial development in the county, industrial wastewater discharge increases annually; therefore, a sewage treatment plant needs to be established so that the wastewater can be processed and reach the discharge standard. Three optional sites around the county are available, from which one or two sites can be selected. Since the county is located in a flood-prone area, the issue of flood control should not be ignored when building a sewage treatment plant. In addition, some other actual situations of the county should be taken into account, such as, the planned area, the overall layout of principal pipelines, the matched technical process, etc. 

### 3.1. Schemes for Site Selection

Minjiang River runs through the county, which is a resort for holidays, exploration, and recuperation, with abundant tourism resources and national nature reserves. According to the overall sewage quantity of the county, principles of site selection and the specific situation of the county industrial development, the designed process capability of a sewage treatment plant is between 20,000 and 50,000 m^3^/day. Considering both the construction scale and the surrounding areas, the planning area of the plant is approximately 10,000–50,000 m^3^. Through a preliminary survey conducted by relevant personnel in the planning process and a solicitation of opinions from involved parties, three relatively satisfactory plans for the location were selected in accordance with the principles and requirements of constructing a sewage plant. The details are as follows:

Scheme I: A sewage plant can be built on a mountain slope with a gradient of 40°. Hence, the land requires leveling; thus, the capital cost of earthwork is evaluated as 190 thousand USD.
Scheme II: A sewage plant can be built on a river bank with a floodwall, which is used for resolving a flood issue. Due to the reinforced concrete structure of the floodwall, a total of 4800 m^3^ of reinforced concrete is needed. Calculating the comprehensive cost of the reinforced concrete at 63.49 USD/m^3^, the required investment is 304.76 thousand USD.
Scheme III: A sewage plant can be built on a river bank without a floodwall. In this situation, the processing equipment needs to be adjusted. It is worth noting that this scheme adopts an ICEAS process (an improved SBR technique). Adopting this process can completely avoid the flood control drawbacks of the aeration equipment. Before the flood, we need only to move the ordinary movable elements without worrying about the key equipment in the sewage plant.

All of the capital expenditures of a sewage treatment plant in the schemes above are illustrated in Table 1. In addition, the proposed reuse rate of the reclaimed water of all schemes is 30%, and the economic benefit of the reclaimed water per unit is 0.19 USD/m^3^.

### 3.2. Influent and Effluent

For the convenience of calculation, the paper assumes that the influent concentration of the sewage source is consistent in this area. Because domestic wastewater accounts for a large percent of the county sewage, and the industrial wastewater belongs to general industrial sewage, COD and NH_3_-N are considered the major pollutants. The influent qualities of sewage water pouring into the treatment plant in this area are determined as:

COD: 252 mg/L NH_3_-N: 35 mg/L

According to the river system distribution and the sewage treatment plant, the treated water ultimately flows into Minjiang River and its tributaries. In accordance with GB18918-2002 “Pollutant Discharge Standard for Municipal Sewage Treatment Plants” primary standard B, the effluent qualities of the treatment plant are required as follows:

COD: ≤60 mg/L NH_3_-N: ≤15 mg/L

The proposed influent and effluent quality and the pollutant removal rate of the treatment plant are shown in Table 2.

### 3.3. Processing Technology

**Figure 3:**
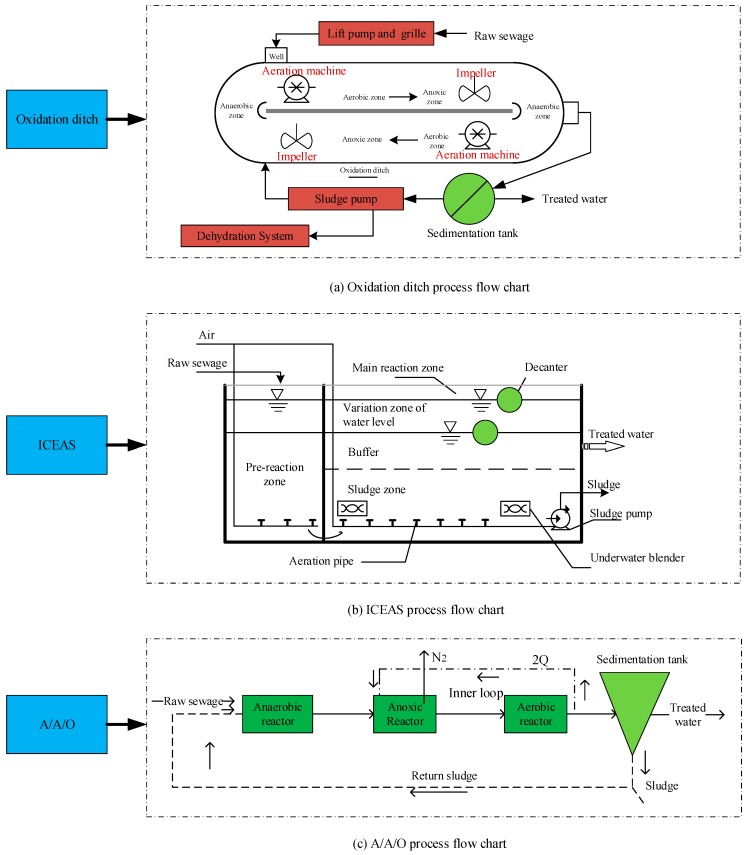
Three types of sewage treatment process flow charts.

Based on the designed processing scale of a sewage plant, the water characteristics, environmental function and the local actual situation and requirements, the sewage treatment process can be selected subject to comprehensive technical and economic constraints. According to the requirements of the influent and effluent quality in this county, as well as the actual situation of Chengdu area, this research considers the following three optional schemes, namely, the oxidation ditch, ICEAS and A/A/O method as the alternatives (see Figure 3). 

Table 3 lists the structures of oxidation ditch, ICEAS, and A/A/O and shows that some structures are consistent, while some are different. Table 4 presents the required equipment of the different structures. 

To summarize, the difference in the equipment costs of the same-size sewage plants primarily lies in the diverse pieces of equipment of a biological treatment unit structure. Therefore, we integrate the cost in terms of equipment when establishing the model. 

### 3.4. Total Costs and Solution Procedure

The total cost of a sewage treatment plant consists of an initial investment cost and an operation cost in this study. In addition, the specific forms of the initial investment cost and the operation cost in practical calculation are different. 

The initial investment cost of a sewage treatment plant in this case comprises costs mentioned in Table 1 and the diverse process investment cost in terms of different techniques. For the convenience of calculation, initial investment cost models of three kinds of techniques were borrowed from other literature and were utilized in this case, as shown in Table 5. 

In addition, the operation cost of a sewage plant refers to the expenditure for sewage treatment after the completion of the project. In the operation of a sewage plant, the operating cost mainly focuses on pipeline maintenance, energy consumption, equipment repair and so forth. In addition to the operation cost listed in Table 6, the sewage treatment costs from different processes are also included.

**Figure 4:**
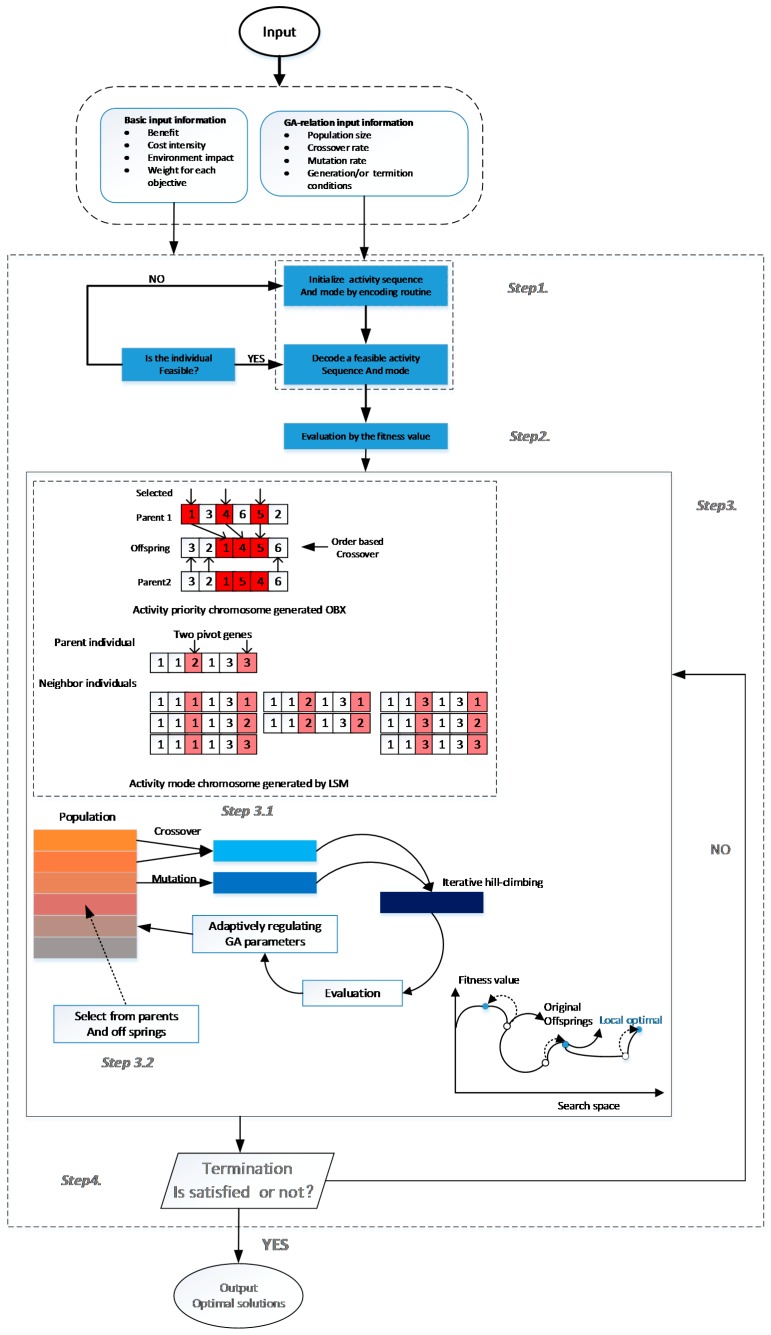
Basic Steps of the Genetic Algorithm.

In this paper, the proposed model is solved by a heuristic algorithm, the Genetic Algorithm (GA), because it is challenging to solve directly. Figure 4 and Table A1 illustrate the basic process of the applied genetic algorithm.

### 3.5. Result Analysis

The parameters for the GA in this problem are as follows: crossover rate 0.2, mutation rate is 0.4, population size is 25 and maximum generation is 500.

Assign weight to the four objective functions in turn:

$$\omega_{1}=0.7,\;\omega_{2}=0.1,\;\omega_{3}=0.1,\;\omega_{4}=0.1$$

Through calculation, the optimal result of the multi-objective model in this situation is:

$$x_{1}\;=\;x_{2}=\;0,\;x_{3}\;=\;1,\;Q_{3}\;=\;3.7\times 10^{4},\;v_{ou3}^{a}\;=\;35.63,\;v_{ou3}^{b}\;=\;2.39$$

Accordingly, under the optimal solution, the minimum cost is 4.19 × 10^6^ USD; the maximum economic benefit is 2.17 × 10^4^ USD; and the removal rates of COD and ammonia nitrogen are approximately 85.1% and 93.2%, respectively. Because *x*_1_ = *x*_2_ = 0, which means that schemes I and II are not considered, any arbitrary value assigned to neither the design capacity nor the removal rate of COD and ammonia nitrogen in the model is meaningless. 

The results above show that within the planned area of 10,000–50,000 m^2^, to achieve such goals as reaching the designed capacity of 20,000–50,000 m^3^/day and meeting the requirements of minimum investment cost, Scheme III should be selected. In Scheme I a method of building a highland sewage treatment plant is adopted, leading to larger investment in earth work and annual operation cost. Through analysis, the paper concludes that Scheme I is not economically feasible. In Scheme II, the sewage plant is surrounded by a flood wall, resulting in a series of problems, such as a large investment and landscape affects. Scheme III adopts provisions to install submersible sewage pumps and jet aeration devices, plan a control room and install the electric transformation and distribution equipment with localized elevation in the building of the sewage plant. Although the investment increases to a certain extent, the overall increase is not too large. Scheme III, therefore, is technically feasible and economically rational.

In terms of techniques, although the improved SBR technique with an ICEAS process is only adopted in Scheme III, it can effectively dispose of pollutants in wastewater and reduce their current content, thereby meeting the requirements of the total discharge control of pollutants. This is attributable to the principal sewage in this area being composed of domestic wastewater, the biodegradability of which is relatively good; meanwhile, the ICEAS process is the secondary biochemical treatment, enabling the COD and ammonia nitrogen removal rate to reach a higher level at 85.1% and 93.2%, respectively. In contrast, even though the optional processes in schemes I and II can achieve the desired effect discussed in the preceding, they are restricted economically due to their higher construction and operation costs.

From the perspective of environmental protection, Scheme III is also based on realistic considerations. The COD and ammonia nitrogen concentrations of the treated sewage are calculated as 35.63 mg/L and 2.39 mg/L, respectively, completely meeting GB18918-2002 “Pollutant Discharge Standard for Municipal Sewage Treatment Plants” primary standard B. Additionally, this scheme locates the treatment plant on the river bank without a flood wall surrounding it. In accordance with the normal requirements, the structural foundation is laid without causing irreversible damage to the surrounding environment (including water, groundwater, cultivated land, forests, aquatic products, landscape, scenic spots, nature reserves, etc.). Moreover, neither is it located in the upper wind zone of urban or residential areas, nor upstream of an urban water resource; therefore, the treatment plant will not affect the residents’ normal life.

According to the importance attached to each target by the decision makers, different weights were assigned to each objective function. Table 7 illustrates the value of each objective function for the conditions and the optimal decision results.

Construction cost is a primary factor considered when building a treatment plant; therefore, it is generally assigned a greater weight value. In accordance with the importance attached to reclaimed water benefit and pollutant discharge amount the weight is adjusted. It is manifested from Table 7 that the weight adjustment of the cost and the reclaimed water benefit will significantly affect the decision result, whereas the weight changes of pollutant treatment efficiency or pollutant emission will not substantially affect the decision result. 

Based on the actual situation and the development plan of an area we can adjust the parameters, such as modifying the sewage quantity and the reuse rate of reclaimed water. Through such adjustments, we can set up a new situation, establish different multi-objective decision models for constructing a treatment plant and obtain various corresponding results by calculation. Since some parameters in this case were calculated based on those of other towns with the same levels, the relevant data might not precisely match the corresponding data; therefore, a situation could arise where the adjustment of a certain index will not significantly affect the decision result, in contradiction with the real situation. Therefore, decision makers should fully understand the specific characteristics of their practical application because the accuracy of the data determines the accuracy of the model. In the practical applications, however, researchers can adjust this model to a certain extent and use this flexibility with actual conditions that can better reflect the real conditions of a given problem domain.

## 4. Conclusions

Considering multiple factors affecting the construction of sewage treatment plants, this paper developed a general multi-objective, decision-based optimizing model for the sewage treatment plant construction plan. In the proposed model, COD and ammonia nitrogen were characterized by random variables because their inherent uncertainty precluded defining them as crisp values or members of only one set. In the case study, the results showed that within the planning area of 10,000–50,000 m^3^, the ICEAS process that was adopted was able to reach the design capacity of 20,000–50,000 m^3^/day and meet the requirements of minimum investment cost, etc. The main conclusions of this study were as follows:

1. This paper introduced the sewage treatment plant construction program’s related issues, specifically describing the existing problems of the sewage treatment plant construction and combining an actual analysis of the sewage treatment plant construction factors. This process identified the key link (the sewage treatment process) in the construction of a sewage treatment plant.
2. Using the framework of uncertainty, random variables were used to characterize COD and ammonia nitrogen, which reduced information losses or distortions while relieving the decision makers’ burden.
3. With the construction of a sewage treatment plant in a suburb of Chengdu as an example, this paper empirically tested the general multi-objective decision model for sewage treatment plant construction. The results verified the applicability and effectiveness of the proposed model and provided decision makers with technical support for the optimization of a sewage treatment plant construction plan.

Last but not least, the weights of each objective were adjusted to account for more information on the construction of the sewage treatment plant and treatment technology selection, and the results showed that changing the weights of the cost and the reclaimed water benefit will significantly affect the decision result. There were many non-modeling activities in designing a solution for the construction of sewage treatment plants, such as the decision-making problem identification and the analysis and assessment of the results, which required the decision-makers’ experience and knowledge and highlights limitations in articulating this practical knowledge; therefore, additional uncertainty in this area can be expected. However, further research can take into account the various complex uncertainties in the decision-making process, and enable the construction of uncertain variables that can be implemented in an uncertainty-based, multi-objective decision-making model.

## Figures and Tables

**Table 1:** Cost schedule of all schemes.

Cost Items	Scheme I	Scheme II	Scheme III
Occupied land (10,000 m^2^)	2.4	4.6	3.5
Land expropriation cost (10,000 USD)	28.57	38.10	34.92
Demolition cost (10,000 USD)	14.29	23.81	20.63
Initial capital cost (10,000 USD)	50.79	47.62	57.14
Sewage pipeline construction cost (10,000 USD)	15.87	31.75	23.81
Sewage pump station construction cost (10,000 USD)	23.81	7.94	15.87
pipeline maintenance cost (10,000 USD)	0.63	0.95	0.63
Pump station operation cost (10,000 USD)	4.13	2.86	3.81
Other operation cost (10,000 USD)	2.54	1.59	1.90
Double circuit return pipe installation cost (10,000 USD)	19.05	15.87	14.29

**Table 2:** The designed influent and effluent quality and pollutant removal rate of the sewage plant.

Item	pH	COD	NH_3_-N
Influent quality (mg/L)	6–9	252	35
Effluent quality (mg/L)	6–9	60	15
Process rate (%)	—	76	57

COD: Chemical Oxygen Demand.

**Table 3:** Comparison between three types of sewage treatment process structures.

Treatment Process	Oxidation Ditch	ICEAS *	A/A/O
Same structures	Coarse screen wells and pumping station, fine screen and grit chamber, blower room, sludge tank, dewatering room, instruments and center control room		
Different structures	Oxidation ditch biological reaction tank, return sludge pump room	ICEAS reaction tank	A/A/O biological reaction tank, secondary sedimentation tank, return sludge pump room

* ICEAS: Intermittent Cycle Extended Aeration System.

**Table 4:** Comparison between three types of sewage treatment process equipment.

Treatment Process	Oxidation Ditch	ICEAS	A/A/O
Equipment	Surface aerator, rotating disc aerator, underwater agitator, submersible axial pump	Micro porous aeration device, plug-flow agitator, water decanter, ICEAS submersible sewage pump	Underwater agitator, underwater propeller, aerator, rotating door, submersible sewage pump, mud scraper, electric hoist, excess sludge pump

**Table 5:** Initial investment cost of three types of sewage treatment processes (lv 2009 [37]).

Treatment Process	Oxidation Ditch	ICEAS	A/A/O
Initial Investment Cost	$C_{1}^{\prime }=-0.0036Q_{i}^{2}+819.13Q_{i}+10^{6};C_{2}^{\prime }=-0.0009Q_{i}^{2}+692.12Q_{i}+2\times 10^{6};C_{3}^{\prime }=-0.0079Q_{i}^{2}+1085.9Q_{i}+2\times 10^{6}$		

**Table 6:** Operation costs of three types of sewage treatment process (lv 2009 [37]).

Treatment Process	Oxidation Ditch	ICEAS	A/A/O
Operation cost	$F_{1}=1.8177Q_{i}^{1-0.0534}$	$F_{2}=1.4113Q_{i}^{1-0.0253}$	$F_{3}=-9E-12Q_{i}^{3}-1E-06Q_{i}^{2}+1.1188Q_{i}$

**Table 7:** Optimal decision results under different weight conditions (variable unit in the table is the same as that in this paper).

$\omega_{1}$	$\omega_{2}$	$\omega_{3}$	$\omega_{4}$	$f_{1}$	$f_{2}$	$f_{3}$	$f_{4}$	$x_{i}$	$y_{ij}$	Q	$v_{\mathrm{out}i}^{\alpha }$	$v_{\mathrm{out}i}^{\beta }$
0.7	0.1	0.1	0.1	0.42	0.22	0.15	0.01	*x*_3_ = 1	*y*_32_ = 1	3.7	35.63	2.39
0.4	0.4	0.1	0.1	0.46	0.29	0.14	0.01	*x*_1_ = 1	*y*_11_ = 1	4.2	29.58	2.31
0.4	0.1	0.4	0.1	0.42	0.21	0.12	0.01	*x*_3_ = 1	*y*_32_ = 1	3.9	27.39	2.44
0.3	0.1	0.2	0.4	0.43	0.22	0.14	0.01	*x*_3_ = 1	*y*_32_ = 1	3.7	33.22	2.31

## Nomenclature

$T_{j,j=1,2\ldots m}$	The sewage treatment process
$A_{i,i=1,2\ldots n}$	The sites of sewage treatment plant
$Q_{i,i=1,2\ldots n}$	The sewage treatment capacity
$v_{in0}^{\alpha }$ $v_{in0}^{\beta }$	Influent concentrations (COD and ammonia nitrogen)
$v_{outi,i=1,2\ldots n}^{\alpha }$ $v_{outi,i=1,2\ldots n}^{\beta }$	Effluent concentrations (COD and ammonia nitrogen)
$B_{i,i=1,2\ldots k}$	The main distributions of sewage source around the treatment plant
$q_{i,i=1,2\ldots k}$	The sewage volumes of each sewage source
$C_{i}(Q_{i},T_{j}),i=1,2\ldots n;j=1,2\ldots m$	Investment costs of the plant construction
$L_{i}(l_{i}),i=1,2\ldots n$	The cost of off-site pipe network
$l_{i,i=1,2\ldots n}$	The length of the pipeline
$D_{i}(s_{i}),i=1,2\ldots n$	The costs of land expropriation and household demolition compensation
$s_{i,i=1,2\ldots n}$	The occupied area of the sewage treatment plant
$\lambda_{i,i=1,2\ldots n}$	The reuse rates of reclaimed water
$E_{i,i=1,2\ldots n}$	The economic benefits per unit reclaimed water
$F_{i}(T_{j}),i=1,2\ldots n;j=1,2\ldots m$	The management operation cost
$\delta_{i,i=1,2\ldots n}$	The score value of the optional site i for the decision makers

## Appendix A

**Table A1:** The GA algorithm for the sewage treatment selection.

Procedure: The GA Algorithm for the Sewage Treatment Selection
**Input:** Initial data and GA parameters
**Output:** Best solutions and objective values
**Step 1.** Initialization. Encode the decision variables and randomly generate an initial population;
**Step 2.** Evaluation. Decode a feasible activity sequence and calculate the fitness function;
**Step 3.** Upgrade. Select the best set of solution from the current generation population and offspring populations;
**Step 3.1** Offspring generation. Fulfill crossover and mutation * and produce offspring solutions;
**Step 3.2** Evolution. Compare the value of newly obtained individual fitness function with those contained in the previous set. Replace the solutions with the new solutions if their value is higher;
**Step 4** Stopping criterion. The algorithm ends when a maximal number of generations is reached, and the best solution, together with the corresponding objective functions, are given as output.

* The mutation operator maintains the diversity of the population and increases the possibility of not losing any potential solution while finding the global optimal solution. The crossover operator is a technique used for rapid exploration of the search space.
